# “HOPE-FIT” in Action: A Hybrid Effectiveness–Implementation Protocol for Thriving Wellness in Aging Communities

**DOI:** 10.3390/jcm14186679

**Published:** 2025-09-22

**Authors:** Suyoung Hwang, Eun-Surk Yi

**Affiliations:** Department of Exercise Rehabilitation & Welfare, Gachon University, Incheon 21936, Republic of Korea; harriett0059@gachon.ac.kr

**Keywords:** hybrid aging model, RE-AIM, implementation science, ACT, community-based intervention, digital health equity

## Abstract

**Background/Objectives:** As global aging accelerates, there is a pressing and empirically substantiated demand for integrated and sustainable strategies, as evidenced by the rising prevalence rates of chronic conditions, social isolation, and digital exclusion among older adults worldwide. These factors underscore the urgent need for multidimensional interventions that simultaneously target physical, psychological, and social well-being. The HOPE-FIT (Hybrid Outreach Program for Exercise and Follow-up Integrated Training) model and the SAGE (Senior Active Guided Exercise) program were designed to address this need through a hybrid framework. These programs foster inclusive aging by explicitly bridging digitally underserved groups and mobility-restricted populations into mainstream health promotion systems through tailored exercise, psychosocial support, and smart-home technologies, thereby functioning as a scalable meta-model across healthcare, community, and policy domains. **Methods:** HOPE-FIT was developed through a formative, multi-phase process grounded in the RE-AIM framework and a Hybrid Type II effectiveness–implementation design. The program combines professional health coaching, home-based and digital exercise routines, Acceptance and Commitment Performance Training (ACPT)-based psychological strategies, and smart-home monitoring technologies. Empirical data from pilot studies, large-scale surveys (N = 1000), and in-depth user evaluations were incorporated to strengthen validity and contextual adaptation. Culturally tailored content and participatory feedback from older adults further informed ecological validity and program refinement. **Implementation Strategy/Framework:** The theoretical foundation integrates implementation science with behavioral and digital health. The RE-AIM framework guided reach, fidelity, and maintenance planning, while the Hybrid E–I design enabled the concurrent evaluation of effectiveness outcomes and contextual implementation strategies. Institutional partnerships with community centers, public health organizations, and welfare agencies further facilitated the translation of the model into real-world aging contexts. **Dissemination Plan:** The multi-pronged dissemination strategy includes international symposia, interdisciplinary academic networks, policy briefs, localized community deployment, and secure, authenticated data sharing for reproducibility. This design facilitates evidence-informed policy, empowers practitioners, and advances digital health equity. Ultimately, HOPE-FIT constitutes a scalable and inclusive model that concretely addresses health disparities and promotes active, dignified aging across systems and disciplines.

## 1. Introduction

As the global population ages at an unprecedented rate, promoting physical activity among older adults has emerged as a key public health priority. Physical activity is essential for maintaining functional independence, improving mental well-being, and preventing or managing chronic diseases in aging populations. However, fostering long-term engagement in physical activity remains a formidable challenge. Structural and psychological barriers—such as mobility limitations, digital exclusion, social isolation, and fragmented caregiving systems—continue to hinder sustained participation.

In a nationally representative sample of Korean older adults (≥65 years), 41.5% were classified as engaging in low levels of activity, according to the International Physical Activity Questionnaire (IPAQ) scoring system, indicating substantial room for improvement in terms of physical activity engagement [1]. Complementing these prevalence data, analyses of 3573 older adults from the same survey demonstrated that higher levels of physical activity were consistently associated with a lower prevalence of sarcopenia, osteoporosis, obesity, and cardiometabolic diseases, as well as more favorable body composition and lipid profiles [2]. Together, these findings underscore that insufficient physical activity in later life is not only highly prevalent but is also strongly associated with multiple age-related health risks.

In parallel, recent Korean studies show high ownership of mobile devices but limited use of wearable and digital health services among older adults, underscoring a persistent functional digital divide that constrains access to health-promoting programs [3]. These patterns point to a clear, evidence-based need for integrated and sustainable strategies that simultaneously address physical inactivity and digital access barriers in aging populations.

To address these multifaceted constraints, various nations have recently adopted hybrid intervention models. Culos-Reed et al. implemented the EXCEL (Exercise for Cancer to Enhance Living Well) model using a hub-and-spoke framework for cancer survivors, offering remote behavior change interventions through coordinated healthcare–community exercise networks [4]. Other initiatives have shown promise; these include the OTAGO and Vivifrail programs for fall prevention in older adults [5,6], the “On the Move in the Community” study utilizing a Hybrid Type I design to evaluate the fidelity and effectiveness of community-based group exercises [7], and Indonesia-based trials of remote rehabilitation via digital home-exercise delivery [8].

Nevertheless, most existing interventions remain narrowly focused—either on specific clinical populations or on single domains such as medical care, exercise, or technology. These approaches often fail to integrate smart-home technology and holistic wellness principles or to build a sustainable, community-anchored health ecosystem for older adults.

To bridge this gap, our research team has spent the past five years developing a context-specific hybrid health model tailored to older adults in South Korea: the HOPE-FIT (Hybrid Outreach Program for Exercise and Follow-up Integrated Training) model. This system transcends conventional single-domain interventions by integrating in-person exercise programs, remote online sessions, and home visits by rehabilitation specialists into a cohesive, multimodal structure. HOPE-FIT is designed to link medical, physical, psychological, technological, nutritional, and community-based infrastructures, thus structurally addressing multi-level barriers while promoting autonomy and social connection among older adults.

The HOPE-FIT model is built on a Korean-style hub-and-spoke framework, forming a multimodal spoke network involving a broad range of professionals: healthcare providers (HCPs), smart-home healthcare instructors (SHIs), exercise specialists (ESs), occupational therapists (OTs), physical therapists (PTs), nutritionists (NTs), dental hygienists (DHs), and health psychologists (HPs). These experts collaboratively support behavioral change by linking individuals’ homes with community health systems.

Under this system, the 12-week SAGE (Senior Active Guided Exercise) program offers multicomponent, personalized interventions including aerobic, strength, balance, and flexibility exercises, along with education on health, nutrition, psychological support, and oral care. Personalized feedback and intervention adjustments are provided based on participants’ health status and home environment, supported by real-time monitoring and smart-home data analytics.

A key innovation of this study is the deployment of advanced smart-home healthcare technology, including wearable smart rings, fall detectors, sleep and gait monitors, and EEG devices sourced from specialized providers, alongside mmWave-based radar motion sensors supplied by RANIX Co., Ltd. Installation and regular maintenance are carried out by dedicated technical teams to ensure safe and reliable use. While these technologies enable real-time physiological monitoring, early risk detection, and personalized feedback to enhance self-regulatory capacity, their novelty may also complicate adoption for older adults—an issue we further consider in the implementation stage.

Importantly, the model also fosters inclusive aging by creating opportunities for intergenerational and community-based engagement. Through the “Wello!” platform, older adults not only participate in exercise routines but also join leisure activities guided by young community leaders, bridging generational divides. Vulnerable or socially isolated individuals are supported through the free installation of smart-home systems, while university-based living labs provide regular health checkups and access to advanced diagnostic devices. In addition, senior cafés embedded within the program offer safe communal spaces for social interaction. Together, these features ensure that inclusivity is not merely rhetorical but concretely embedded in both the design and delivery of the HOPE-FIT model.

To evaluate the multidimensional effectiveness and real-world applicability of the HOPE-FIT model, we adopted the RE-AIM framework—assessing reach, effectiveness, adoption, implementation, and maintenance. In this process, we explicitly define “holistic health outcomes” in alignment with the International Classification of Functioning, Disability and Health (ICF). These outcomes extend beyond physical fitness to encompass psychological well-being, autonomy in daily functioning, social participation, and digital literacy—domains that are distinct from but complementary to the program’s specific clinical and behavioral focus areas. This framing ensures that the HOPE-FIT model evaluates aging in a multidimensional manner, capturing the broader determinants of health and quality of life.

The effectiveness of the SAGE program is evaluated through repeated measurements at three time points (pre-intervention, post-intervention, and follow-up stages), focusing on quality of life, functional fitness, autonomy, social support, and digital literacy. This approach provides insights into both holistic health outcomes and the mechanisms supporting sustained engagement.

Rather than being an intervention merely based on physical activity, HOPE-FIT is a systems-based solution aimed at restoring holistic wellness and social connectedness among older adults. With institutional backing from the Korea Smart Home Healthcare Association, we have developed a national certification and training infrastructure for SHIs, OTs, and NTs to build professional capacity. These efforts aim to strengthen linkages between communities and healthcare systems and to lay the groundwork for the nationwide dissemination of the HOPE-FIT model.

Thus, this protocol presents the theoretical foundations, formative evaluation, and hybrid effectiveness–implementation study design of the HOPE-FIT model. The specific objectives of this study are as follows:

1. To evaluate the feasibility and scalability of the HOPE-FIT infrastructure across diverse community settings.
2. To examine the clinical and behavioral effects of the SAGE program.
3. To provide policy-relevant recommendations applicable to Korea and other aging societies.

## 2. Theoretical Framework

This study applied an integrated theoretical framework combining the RE-AIM model (Reach, Effectiveness, Adoption, Implementation, Maintenance) and the Hybrid Type II Effectiveness–Implementation Design to comprehensively evaluate both the efficacy and feasibility of the HOPE-FIT (Hybrid Outreach Program for Exercise and Follow-up Integrated Training) model. This framework was designed not only to assess intervention outcomes but also to understand how a hybrid community-based health management system can function and be scaled in real-world aging populations.

### 2.1. RE-AIM Framework

The RE-AIM framework, originally proposed by Glasgow and colleagues, offers a multidimensional evaluation model to assess the public health impact of behavioral interventions [9]. In this study, we extended its application beyond conventional integrating digital health indicators, multi-level ecological determinants, and participant–provider communication processes, as follows:

- Reach: Evaluation includes not only the demographic and psychosocial characteristics of older adult participants in HOPE-FIT but also their levels of digital literacy, access to healthcare services, and socioeconomic vulnerabilities.
- Effectiveness: Outcomes such as physical activity level, functional fitness, quality of life, social connectedness, and perceived health status are assessed at baseline, immediately post-intervention, and at a six-month follow-up. Additionally, complementary digital biomarkers and behavioral data—such as sleep patterns, fall detection, and mobility metrics derived from smart-home systems—are used to evaluate effectiveness from a digital health perspective.
- Adoption: Characteristics and participation levels of local institutions (e.g., public health centers, senior welfare centers) and frontline professionals (e.g., HCPs, SHIs) are analyzed, with attention to factors including their willingness to adopt, digital capacity, and perceived implementation readiness. This dimension captures ecological determinants such as cross-institutional collaboration, resource availability, and training efficacy.
- Implementation The fidelity of program delivery, usability of digital interfaces, and participants’ adaptation processes are evaluated using a multi-channel feedback-loop system that includes (i) TIDieR-based fidelity checklists and session logs completed by implementers, (ii) participant-facing micro-surveys embedded in the app (e.g., RPE, pain flags, perceived usefulness), (iii) smart-home and wearable dashboards that summarize safety/engagement signals, and (iv) brief post-visit SHI checklists capturing contextual barriers and adaptations. All feedback streams are routed to a HUB dashboard through a predefined data pipeline, where rule-based thresholds (e.g., ≥2 consecutive high RPE flags, new pain flags, >30% adherence drop) automatically trigger a priority queue for action. Actions such as JITAI messaging, exercise intensity changes, safety calls, or home-visit scheduling are logged in real time, reviewed in weekly huddles, and, if persistent, promoted to protocol updates during monthly PDSA cycles. Data minimization and participant opt-out options are embedded to preserve autonomy while enhancing ecological validity.
- Maintenance: The long-term sustainability of behavioral changes at the individual level and institutionalization of the HOPE-FIT model at the organizational and community levels are assessed. This includes evaluating structural integration into public service networks and continuity planning post-intervention.

By applying this expanded RE-AIM framework, this study seeks to evaluate not only the public health effectiveness but also the scalability and sustainability of technology-integrated hybrid interventions. The structured feedback loops, embedded within the intervention architecture, ensure responsiveness to the lived context of older adults, thereby enhancing ecological validity and real-world adaptability.

### 2.2. Hybrid Type II Effectiveness–Implementation Design

To achieve the dual objectives of evaluating the intervention’s effectiveness and implementation feasibility, the study employed the Hybrid Type II design [10]. This design is particularly closely aligned with the study’s objectives in the following ways:

- Simultaneous Evaluation of Clinical and Implementation Outcomes: The study simultaneously examines clinical and behavioral outcomes—such as functional fitness, perceived health status, and social engagement—alongside implementation dimensions, including practitioner training, institutional readiness, and execution fidelity at the local level (e.g., by HCPs and SHIs).
- Integration of Formative Evaluation: A formative evaluation process is embedded throughout program planning, execution, and adaptation stages, capturing real-time feedback from both implementers and participants to refine intervention strategies and improve contextual fit. For example, if ≥20% of a cohort reports new knee pain flags within seven days, the HUB triggers triage calls within 24 h, swaps the exercise library to a low-impact pre-hab module for two weeks, and initiates HCP audits. If pain flags subside for two consecutive weeks, the original module is reinstated; if not, the adaptation is promoted to a protocol change during the monthly PDSA cycle. Each step is logged and fed back to participants in plain language, exemplifying how formative evaluation operationalizes the Hybrid Type II design while reinforcing ecological validity.
- Operationalization of Bidirectional Feedback Loops: Bidirectional feedback loops are embedded at three levels:
  (1) Participant ↔ Provider: participants send in-app symptom/motivation check-ins and sensor data streams to the HUB; providers respond with just-in-time adaptive interventions (JITAI).
  (2) Provider ↔ Provider (within and across sites): ES/SHI/HCP teams log barriers/adaptations on TIDieR forms, review them in weekly huddles, and share solutions in HUB-led monthly QI cycles.
  (3) System ↔ Implementer: The HUB dashboard applies decision rules to trigger alerts, workflow nudges, and protocol refinements, which are then communicated back to implementers.

Cadence is mixed (daily automated flags, weekly huddles, monthly cycles), and all adaptations are captured in time-stamped change logs, reducing recall bias and ensuring rapid, context-sensitive responsiveness.

- Addressing Digital Aversion, Behavioral Resistance, and Workforce Maldistribution: To address digital aversion, the HOPE-FIT model implements a three-step onboarding pathway: (i) Analogue First (paper guides, phone check-ins), (ii) Assisted Use (loaner devices with navigator support), and (iii) Independent Use (graduated prompts, simplified UI). Caregiver co-use is enabled through secondary log-ins, while ACT-based micro-modules with graded exposure promote gradual acceptance. Implementation metrics include the onboarding completion rate, time-to-independence in app use, ACT module completion, and navigator contact minutes. To address workforce maldistribution, standardized TIDieR packages and HUB-based remote supervision allow novice SHIs to deliver core content with asynchronous review by senior HCPs, supplemented by weekly huddles and monthly cross-site case conferences. When local staffing falls below threshold, predefined fallback modes (tele-prehab, group tele-coaching) ensure service continuity.

**Figure 1:**
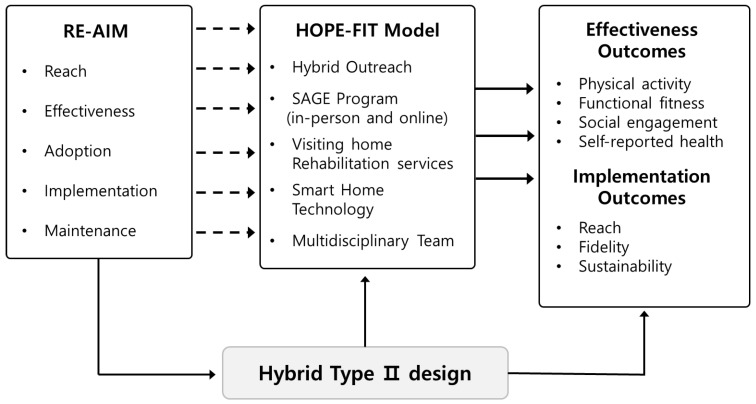
Structure conceptual framework. Conceptual framework of the HOPE-FIT model, illustrating the integration of the RE-AIM evaluation domains with a Hybrid Type II design. The diagram highlights the bidirectional feedback loops between participants, providers, and system implementers, which reinforce ecological validity and real-world adaptability.

Taken together, HOPE-FIT provides a systems-level advance: tri-level feedback loops shorten the signal-to-adaptation interval; JITAI-enabled hybrid delivery with remote supervision decouples reach from local workforce density; and RE-AIM-aligned metrics specify a pathway for institutionalization (see Figure 1). These multi-level mechanisms address entrenched structural barriers in access, capacity, and continuity, positioning HOPE-FIT as a transformative model for aging populations.

### 2.3. Alignment with Study Structure

This theoretical framework is directly aligned with the background and problem statement outlined in the Introduction, and it offers a comprehensive analytical scaffold for addressing the study’s three core objectives: (1) implementation feasibility, (2) intervention effectiveness, and (3) translational and policy-level scalability. The framework is systematically articulated in the Theoretical Framework section, where the integration of the RE-AIM model and the Hybrid Type II Effectiveness–Implementation Design is clearly established.

In the Methods section, the RE-AIM domains are explicitly operationalized across each of the core methodological components:

- Study Design (Section 3.1): This explains the phased approach to implementation and outcome evaluation.
- Participants and Sampling (Section 3.2): This describes the purposive sampling strategy, including the intentional recruitment of digitally marginalized older adults.
- Intervention (Section 3.3): This details the structure of the HOPE-FIT and SAGE programs, emphasizing hybrid delivery mechanisms.
- Evaluation Tools and Outcomes (Section 3.4): This specifies both quantitative indicators (e.g., physical function, quality of life, digital literacy) and biometric data (e.g., sleep, gait, fall risk) directly linked to RE-AIM metrics.
- Data Collection Procedures (Section 4): This reflects the alignment of repeated measurement points with RE-AIM domains.

Notably, the embedded formative evaluation process reinforces the Hybrid Type II design by operationalizing a real-time feedback loop across participants, providers, and system implementers. For example, if ≥20% of participants report new pain flags within one week, the HUB automatically triggers triage calls, swaps in a low-impact pre-habilitation module, and prompts an HCP audit of recorded sessions. Resolution of pain reports over two consecutive weeks results in reinstatement of the original protocol; persistence escalates the adaptation to a protocol change during the monthly PDSA cycle. This mechanism demonstrates how formative evaluation facilitates the iterative refinement of intervention strategies, enhances contextual fit, and strengthens ecological validity in real-world settings.

Ethical safeguards, including informed consent and IRB approval, are addressed in the Ethics section. Furthermore, the Dissemination Plan outlines strategic pathways for knowledge translation, community adoption, and policy integration, ensuring that the research findings are meaningfully scaled and sustained.

## 3. Methods

### 3.1. Study Design

This study adopted a Hybrid Type II Effectiveness–Implementation Design to comprehensively evaluate both the clinical effectiveness and real-world applicability of the HOPE-FIT (Hybrid Outreach Program for Exercise and Follow-up Integrated Training) model. This design provides a robust theoretical framework that enables the simultaneous assessment of individual-level health outcomes and systems-level implementation strategies, making it well-suited for empirically analyzing how a hybrid health management system for older adults operates and diffuses within the community setting [10,11].

Furthermore, the study employed the RE-AIM framework—Reach, Effectiveness, Adoption, Implementation, and Maintenance—to assess the program’s public health impact, acceptability, fidelity, and long-term sustainability using both quantitative and qualitative approaches. The traditional RE-AIM dimensions were extended to incorporate ecological-level structural factors, including digital literacy, inter-agency collaboration, and acceptance of smart-home technology. Key performance indicators (KPIs) were not arbitrarily chosen but were derived from triangulated formative data sources—a nationwide survey (N = 1000), a UI/UX pre-evaluation study (N = 150), and pilot interventions with digitally marginalized older adults. This ensured that the KPIs captured the heterogeneity of older adults’ needs. Measurement consistency was guaranteed by HUB-standardized assessment protocols (e.g., WHOQOL-BREF for QoL, EARS for adherence, Digital Literacy Scale, wearable-based biometrics), while SPOKE sites could prioritize certain indicators but were synchronized through HUB-led Quality Improvement (QI) cycles for comparability.

**Figure 2:**
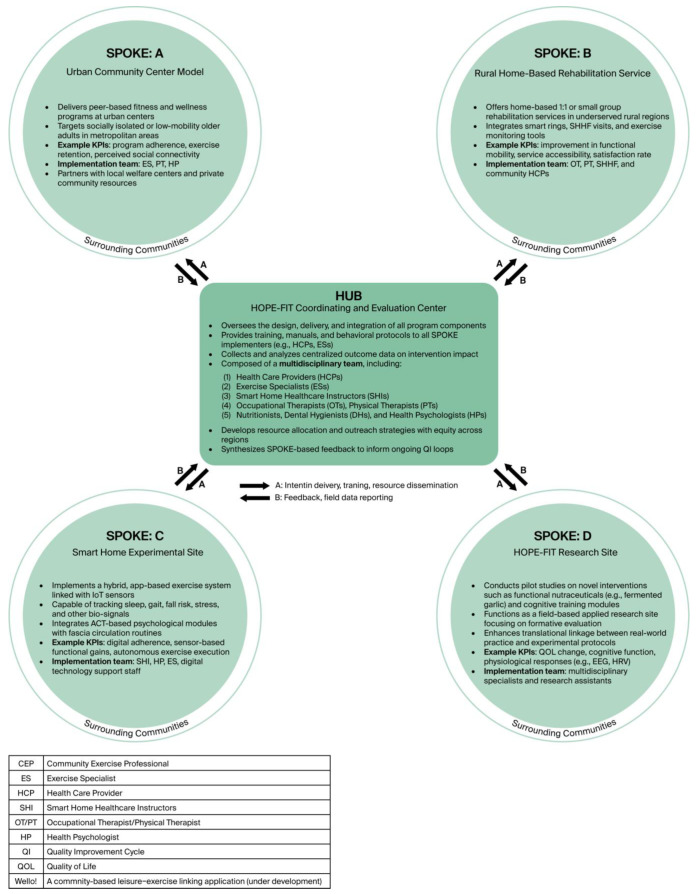
Multimodal spoke network for HOPE-FIT. Multimodal hub-and-spoke network of the HOPE-FIT program. The central HUB coordinates standardized protocols, training, and evaluation, while SPOKE sites (A–D) represent diverse implementation settings such as urban centers, home-based rehabilitation, smart-home experimental sites, and research clinics.

To operationalize this hybrid design, we constructed a multimodal spoke network for exercise rehabilitation across diverse delivery contexts. As illustrated in Figure 2, the HOPE-FIT model was structured around a central HUB, which served as the coordination and evaluation core, and four SPOKE nodes, each representing a unique intervention setting. The HUB provided standardized intervention protocols, educational materials, behavioral components, and technological support to all SPOKE sites and received ongoing feedback through a structured QI cycle. Each SPOKE was designed to address specific contextual needs:

- SPOKE A targeted urban community centers offering fitness sessions, ACT-based counseling, and wearable sensor monitoring.
- SPOKE B delivered home-based rehabilitation with cognitive stimulation and economic incentives.
- SPOKE C functioned as a smart-home experimental site, enabling integrated monitoring and digital assistance.
- SPOKE D supported HOPE-FIT trials and implementation research, including clinician training and usability testing.

Each SPOKE implemented customized programs aligned with multi-dimensional KPIs. The KPI framework was organized into five domains: (1) physical function and autonomy, (2) psychological resilience, (3) social connectedness, (4) digital adaptation, and (5) biometric/behavioral economics. Each was linked to standardized HUB protocols. For example, SPOKE A emphasized quality of life, exercise adherence, and social support, whereas SPOKE C prioritized biometric signals from smart-home devices. All SPOKE-specific KPIs were synchronized through HUB-QI cycles, ensuring adaptability while maintaining methodological comparability.

This diagram visualizes the HOPE-FIT (Hybrid Outreach Program for Exercise and Follow-up Integrated Training) model as a multimodal, community-adaptive framework designed to deliver and scale exercise rehabilitation across diverse settings. The central HUB serves as the coordinating and evaluation node, providing standardized intervention protocols, educational materials, behavioral components, and technological support. Each SPOKE functions as a context-specific intervention unit—targeting urban fitness centers, home-based care, smart-home experimental environments, and research/testing settings—and implements customized programs. Key performance indicators (KPIs) are contextually prioritized but standardized through HUB-QI cycles to ensure both adaptability and comparability.

The study was implemented in the following three phases:

① Formative Phase
  - A 13-item custom-developed survey assessed participants’ health needs, digital literacy, and receptivity to smart-home technology.
  - Focus group interviews (FGIs) were conducted with local implementation institutions and domain experts.
  - An environmental audit of smart-home technology infrastructure and participants’ digital adaptability was performed.
  - A small-scale pilot of the Senior Active Guided Exercise (SAGE) program examined feasibility and contextual suitability.

② Implementation Phase

A 12-week hybrid SAGE program was administered, consisting of the following multi-component interventions, each of which was theoretically justified and adjusted for older adults’ accessibility:

(1) Combined in-person and remote physical activity sessions: These provided structured group-based exercise alongside app-guided routines, ensuring continuity of engagement across settings. This dual modality addressed barriers such as transportation difficulties and health fluctuations.
(2) ACT-based psychological recovery program (8 sessions): These were grounded in ACT principles, targeting psychological flexibility, emotional regulation, and values-based behavior.
(3) Smart home-based health monitoring system: Wearable and environmental sensors (e.g., sleep, gait, heart rate, stress) enabled continuous tracking, reinforcing ecological validity, safety, and personalized feedback.
(4) Acceptance and Commitment Performance Training (ACPT)–Fascia Circulation Exercise: This was developed to promote emotional cleansing and psychophysical integration through myofascial flow.
(5) Behavioral economic strategies: These clarified identity cues and social reinforcers, including branded T-shirts, eco-bags, keyrings, meal vouchers, community incentives, and honoraria. These functioned as visible identity markers, fostering belonging and reciprocity, thereby converting extrinsic nudges into intrinsic motivation for long-term adherence.
(6) Digital literacy education and device adaptation support: Structured stepwise training, simplified interfaces, and navigator assistance reduced digital aversion and bridged the technological divide.
(7) Health-oriented dietary and lifestyle counseling: Nutritionists and allied health professionals provided holistic guidance on diet, sleep, and stress.
(8) Professional rehabilitation services: These were delivered through interdisciplinary collaboration between physical therapists, occupational therapists, and healthcare providers to safeguard fidelity and personalization.
(9) Wearable-based data collection: Smart rings, radar sensors, and electroencephalogram (EEG) devices provided objective feedback, reinforcing participant self-efficacy.

To mitigate complexity and the participant burden, the intervention followed a core + optional model: three weekly peer sessions formed the mandatory core, while app-based routines and smart-home visits were tailored adaptively to participant capacity and preference. Engagement was supported by simplified digital interfaces, caregiver co-use, navigator guidance, and ACT-based coaching.

All components were described in accordance with the Template for Intervention Description and Replication (TIDieR) checklist [12] and classified using the Expert Recommendations for Implementing Change (ERIC) taxonomy [13].

③ Evaluation Phase

Assessments were conducted at baseline, post-intervention, and six-month follow-up, covering the following domains:

(1) Quantitative Evaluation:
  - Quality of life (WHOQOL-BREF), autonomy, exercise adherence (EARS), rehabilitation perceptions, outcome expectancy, social support for exercise, anxiety, depression, and health-information-seeking behavior;
  - Physical function (e.g., 30-s chair stand test, gait speed) assessed onsite by exercise specialists (ES) and physical therapists (PT);
  - Cognitive function measured by neuropsychological experts using MMSE-K, MoCA-K, TMT, CDT, and GDSQ;
  - Smart device–based biometric indicators (e.g., sleep, heart rate, gait patterns, EEG);
  - KPI change rates over time (e.g., functional improvements, digital compliance).
(2) Qualitative Evaluation:
  - Semi-structured in-depth interviews (N=30) on exercise engagement, technology adoption, ACT-based acceptance transformation, and community connectivity;
  - Open-ended questionnaire-based narrative evaluations;
  - Session-wise ACT responsiveness and acceptance evaluated through feedback forms and researcher field notes;
  - Tracking of incentive response patterns.
(3) Digital-Based Evaluation:
  - Self-directed exercise adherence rates and in-app log analysis;
  - Monitoring of program execution rate, digital device utilization, and dropout rates.

Smart devices were retrieved within three days of intervention completion. Real-time alerts were transmitted to a centralized HUB for emergency monitoring, providing a safety net. The biometric data were further utilized to develop personalized algorithmic models for health intervention, suggesting future scalability toward digital health platforms.

This study adopted a community-based participatory approach (CBPA), building cooperative networks with a wide range of experts to enhance both field-level implementation and sustainability. The professional collaborators included healthcare providers (HCPs), smart-home healthcare instructors (SHIs), exercise specialists (ESs), occupational therapists (OTs), physical therapists (PTs), nutritionists (NTs), dental hygienists (DHs), and health psychologists (HPs).

The detailed application of the Hybrid Type II design and RE-AIM framework is summarized in Table 1.

### 3.2. Participants and Sampling

This study employed a Hybrid Effectiveness–Implementation Design with a formative evaluation approach to assess the preliminary structure and feasibility of the Hybrid Outreach Program for Exercise and Follow-up Integrated Training (HOPE-FIT) model. The primary participants were older adults who were exposed to compounded risk factors such as digital exclusion, chronic diseases, and social isolation. Although the conventional threshold for defining “older adults” is often set at 60 or 65 years of age, this study intentionally included individuals aged 50 and above to capture those in the transitional mid-life stage, where preventive interventions may forestall decline and facilitate smoother adaptation to hybrid health programs.

The preliminary phase of model development was structured as a multi-layered and integrative process. In collaboration with EMBRAIN (certified by ISO 20252) [14], a leading survey agency in Korea, a nationwide quantitative study was conducted with 1000 community-dwelling adults aged 50 years and older. The survey examined digital literacy, barriers to physical activity, social isolation, and behavioral economic factors. Stratified sampling by age and region was applied, and a unique ID-based response system was employed to ensure validity and representativeness. To enhance accessibility, surveys were administered in mixed modes (online, paper-based, and interviewer-assisted), with trained staff providing verbal guidance for participants with low literacy, sensory impairments, or cognitive limitations. These findings demonstrated a critical demand for hybrid community-based interventions, underscoring the urgent need for scalable health programs in the context of Korea’s rapidly aging population [15].

To further refine the intervention, a five-month user interface/user experience (UI/UX) pre-evaluation was conducted with 150 older adults aged 65 and above. Thirty of these participants engaged in in-depth interviews and focus group discussions to explore exercise motivation, usability, and health information literacy. Using the analytic hierarchy process (AHP), priority needs for health promotion and intervention strategies were quantitatively derived and directly integrated into the program design.

A series of foundational pilot studies also tested preliminary program elements. Despite low baseline motivation, weak social connectedness, and financial barriers, participants demonstrated high willingness and sustained adherence to peer-based exercise systems. These findings directly informed the behavioral economic strategies adopted in the present study, including branded T-shirts, modest honoraria, and digital literacy reinforcement.

**Figure 3:**
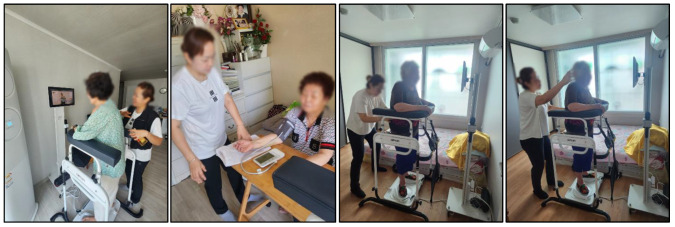
On-site image of home-based smart healthcare rehabilitation visit. On-site image of a smart-home healthcare instructor (SHI) conducting a home-based rehabilitation visit. The visit included device setup, biometric monitoring, and individualized coaching to reinforce adherence and safety in the home environment.

In the pilot phases (2021–2023), participants with severe chronic diseases or medical conditions that could pose safety risks during exercise were excluded to ensure feasibility testing in a controlled population. This methodological decision was necessary to validate the protocol’s safety and implementation structure before expanding to more vulnerable groups. However, in the ongoing smart-home extension studies (2024–2025), recruitment was broadened to include older adults with selected chronic conditions and socially vulnerable populations, such as those at risk of falls or experiencing digital exclusion. For these participants, tailored interventions were implemented, including the use of the DIPDA fall-prevention device (see Figure 3, wearable diaper-type monitoring equipment, and culturally adapted Trot-dance-based exercise sessions delivered by home-visit instructors. This phased approach reflects the multi-year design of HOPE-FIT: beginning with feasibility in healthier cohorts and progressively scaling to inclusivity for frail and chronically ill populations.

The hybrid intervention was implemented in a phased structure, aligned with the Reach and Implementation domains of the RE-AIM framework:

- **March–September 2022:** A pilot with 25 participants tested the feasibility and effectiveness of the hybrid exercise model. The recruitment rate was 92%, and 23 participants completed the program (92% retention). Preliminary results indicated improvements in functional strength (average +14% increase in chair-stand test) and reduced self-reported fatigue. Barriers and facilitators were explored through in-depth interviews and participant observation.
- **March 2023–March 2024:** A one-month hybrid intervention was conducted with approximately 50 older adults. The recruitment rate was 88%, with 44 participants completing the program (88% retention). This phase incorporated Acceptance and Commitment Performance Training (ACPT)-based fascia circulation exercises, black garlic intake (supported by preliminary evidence for antioxidant and fatigue recovery effects), and cognitive stimulation activities. Preliminary effects included significant improvements in digital literacy (+11% on the Digital Literacy Scale) and self-reported quality of life (+9% on the WHOQOL-BREF). The total intervention period exceeded 12 months.
- **April 2025–ongoing:** A comprehensive hybrid intervention is now being implemented with 60 older adults. Recruitment has been completed, with an interim retention rate of 95% at Week 6. Participants are engaging in ACPT-based fascia circulation exercises, cognitive stimulation tasks, Acceptance and Commitment Therapy (ACT)-based psychological recovery sessions, and smart-technology-assisted health monitoring. Interim monitoring indicates >80% compliance with app-based exercise logging and strong adherence to smart-home monitoring protocols. Written informed consent was obtained from all participants prior to enrollment.

The hybrid program combined peer-based group exercise sessions with an app-based adaptation protocol and incorporated multiple healthcare domains, including:

- **Smart-home technologies:** Smart rings, radar sensors, fall detection devices, gait/sleep/mental health monitoring, electroencephalogram (EEG)-based neurofeedback, and transcutaneous vagus nerve stimulation (tVNS).
- **Psychological sessions:** ACT-based structured acceptance interventions and tactile/cognitive stimulation activities.
- **Behavioral economic incentives:** Branded T-shirts, small honoraria, and repeated digital literacy reinforcement, strategically designed as identity cues and low-cost motivators for sustained adherence.
- **Embodied psychomotor interventions:** ACPT-based fascial circulation exercises to enhance somatic–cognitive integration and myofascial flow.

Recruitment was conducted purposively through local health centers, welfare agencies, and community centers. Recruitment rates and representativeness were monitored according to the Reach dimension of the RE-AIM framework.

**Figure 4:**
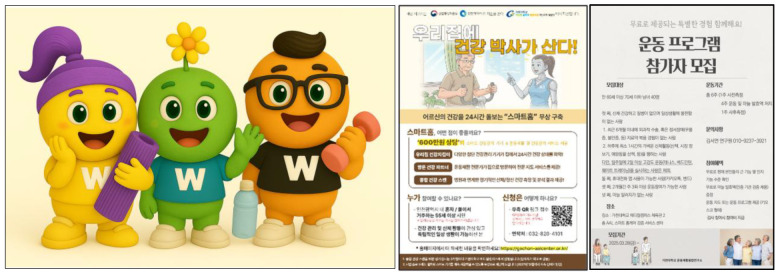
Wello! app character design and participant recruitment brochure from multi-year study. Wello! platform design and participant recruitment materials used in multi-year pilot studies. The figure shows the culturally tailored character design and outreach brochures that were employed to enhance recruitment, engagement, and digital inclusivity among older adults. https://play.google.com/store/apps/details?id=com.gachoncrf.wello&pcampaignid=web_share (accessed on 17 September 2025).

To ensure sustainability, the research team is developing a community-integrated mobile application platform, Wello! to promote leisure–exercise participation and wellness tourism among older adults. This platform, including its culturally tailored app character design and participant recruitment materials, is envisioned as a long-term maintenance strategy to consolidate behavioral gains within the HOPE-FIT framework (see Figure 4). Furthermore, expansion into rural areas is underway, with the long-term goal of scaling the HOPE-FIT model across six regional zones to establish a locally adaptive integrated public health infrastructure and validate national-level scalability.

### 3.3. Intervention: Hope-FIT and SAGE Program

The intervention applied in this study was based on the Hybrid Outreach Program for Exercise and Follow-up Integrated Training (HOPE-FIT) model and implemented through the Senior Active Guided Exercise (SAGE) program. Designed as a multimodal hybrid health intervention, SAGE aims to promote physical activity and holistic well-being among older adults by integrating movement, psychological strategies, digital healthcare technologies, and home-based outreach services.

**Figure 5:**
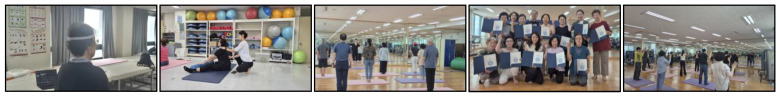
ACPT-based fascia circulation exercise session. Example of an ACPT-based fascia circulation exercise session delivered during peer-based group meetings. The image demonstrates how Acceptance and Commitment Performance Training principles were integrated with fascia-focused physical movements to enhance mind–body integration.

The intervention structure consisted of the following weekly components:

**Three Weekly Peer-Based In-Person Group Sessions**
  - Location: Wello! Center or Active Aging Lab (AAL) Center at Gachon University.
  - Facilitators: Certified exercise specialists (ES) and health psychologists (HP).
  - Composition: Each session integrated fascia-based circulation exercises and Acceptance and Commitment Therapy (ACT)-based psychological recovery tasks. (see Figure 5)
  - Objective: To enhance peer motivation, emotional bonding, and social connectedness among participants.

**Two Weekly App-Based Self-Guided Sessions**
  - Delivery: Digital content was delivered via the app, including structured movement routines, psychological missions, and adaptive reminders.
  - Participant Activity: Participants completed sessions at home and logged exercise performance and self-reflections through the app interface.
  - Objective: To foster autonomy, habitual engagement, and reinforcement of behavior.

**Two Weekly Smart Home Healthcare Visits**
  - Personnel: Smart-home healthcare instructors (SHIs) conducted home visits.
  - Monitoring Tools: Smart rings, radar sensors, gait/sleep/stress tracking systems, electroencephalogram (EEG) devices, and transcutaneous vagus nerve stimulators (tVNS).
  - Concurrent Activities: Cognitive stimulation tasks and digital literacy education were embedded within the visit.
  - Objective: To cultivate tech-friendly self-care habits and support sustained behavior through real-time data feedback and coaching.

**Figure 6:**
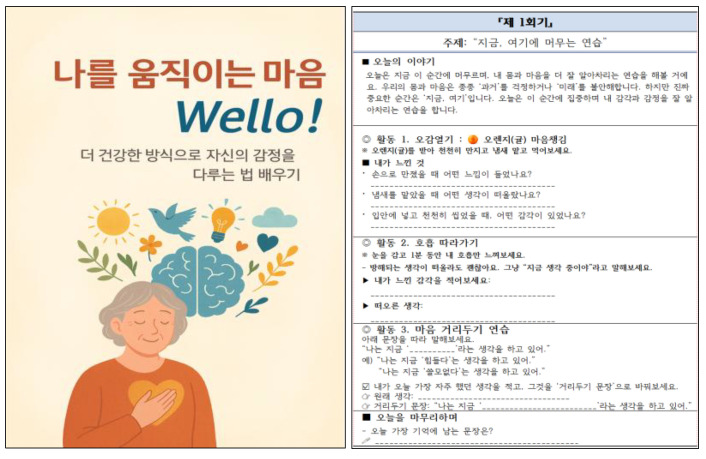
Session 1 excerpt from the eight-session ACT-based psychological workbook. Excerpt from Session 1 of the eight-session ACT-based psychological workbook used in the SAGE program. The workbook provides structured guidance on emotional regulation, value clarification, and behavior commitment tailored to older adult participants.
  2. **Fascial Circulation Exercises with ACT Principles**
    Unlike conventional physical activity programs, these exercises incorporated mindful awareness of bodily sensations and acceptance of present-moment experience. Built around principles such as rolling, gliding, bouncing, stretch–hold–wave, and breath–release, they were designed to improve fascial elasticity, enhance postural control, relieve pain, and promote psychological relaxation simultaneously.
  3. **Smart Health Feedback System**
    Biometric data (e.g., sleep quality, gait, heart rate) collected via wearable and environmental sensors were synthesized with app-based information to generate personalized health feedback. Automated reminders and motivational messages were delivered, while quality improvement (QI) cycles grounded in the RE-AIM framework ensured fidelity and long-term impact.
  4. **Behavioral-Economics-Based Incentive System**
    Incentives such as branded T-shirts, eco-bags, app-based mileage rewards, and peer recognition were implemented not as superficial rewards but as identity cues and social reinforcers. Drawing on behavioral economics, these low-cost high-impact nudges fostered a sense of belonging and achievement, thereby supporting long-term adherence and the conversion of extrinsic motivation into intrinsic motivation.
  5. **SHI Training and Personalization Procedures (TIDieR-based interventions)**
    Smart-home healthcare instructors (SHIs) underwent a standardized training curriculum accredited by the Korea Smart Home Healthcare Association, including modules on exercise safety, digital literacy support, motivational interviewing, and culturally tailored communication strategies. Training fidelity was ensured using TIDieR-based checklists. Personalization of exercise and digital coaching was guided by baseline assessments of functional fitness, cognitive status, and digital literacy. During each home visit, SHIs adapted the content by adjusting exercise intensity, selecting appropriate digital tools (e.g., simplified vs. advanced app interface), and providing caregiver co-use guidance when necessary. Continuous micro-feedback from wearable sensors and participant self-reports enabled iterative refinement, ensuring that each participant received an individually tailored intervention. In addition, SHIs received specialized education and certification training on the use of the DeepDa device, a dance-based exercise tool incorporating fall-prevention support functions. This training covered not only device operation but also comprehensive coursework in basic anatomy, pathology of age-related diseases, and exercise prescription principles. SHIs were trained to design and operate programs tailored to different levels of physical function, age groups, and disease conditions. Practical training sessions were complemented by structured practicum requirements, during which SHIs documented their field experiences through detailed training logs and reflective practice journals. These educational and experiential components ensured that SHIs were fully prepared to deliver safe, evidence-based, and personalized interventions across diverse older adult populations.

**Summary of Integrated Components**
  1. **ACT-Based Psychological Intervention**
    The psychological component was grounded in the ACT model, emphasizing emotional regulation, value clarification, and commitment-based behavior change. These methods are particularly relevant to older adults, who frequently experience motivational decline and psychosocial barriers (see Figure 6).

**Rationale for Hybrid Complexity and Simplification Strategies**

Although the HOPE-FIT intervention integrates multiple components (peer-based sessions, app-guided routines, and smart-home visits), this complexity was deliberately designed to reflect the heterogeneity of older adults’ health conditions, digital readiness, and social connectedness. Importantly, participation followed a modular “core-plus-optional” model: three weekly peer-based sessions functioned as the mandatory foundation, while app-based and smart-home components were selectively provided according to individual capacity and preference.

To minimize burden, the program employed gradual onboarding, simplified digital pathways, and family co-participation options. Engagement was reinforced through peer support networks, HUB-based monitoring of adherence and safety, and identity-based incentives that combined social recognition with symbolic reinforcement.

Thus, the hybrid complexity enhances feasibility, inclusivity, and ecological validity, while built-in simplification strategies ensure that the program remains accessible, sustainable, and adaptable across diverse community settings

### 3.4. Evaluation Tools and Outcomes

This study employed a multidimensional evaluation framework to assess the effectiveness, implementation feasibility, and sustainability of the Senior Active Guided Exercise (SAGE) program based on the Hybrid Outreach Program for Exercise and Follow-up Integrated Training (HOPE-FIT) model. The evaluation was guided by the RE-AIM framework (Reach, Effectiveness, Adoption, Implementation, and Maintenance) and integrated quantitative, qualitative, and digital assessment tools, alongside smart-home-based bio signal and environmental monitoring data. This comprehensive approach was designed to move beyond simple pre/post comparisons, capturing diverse domains such as behavioral change, psychological resilience, digital acceptance, and social connectivity.

The evaluation framework was explicitly aligned with RE-AIM domains, whereby the quantitative tools primarily assessed effectiveness, the qualitative tools informed adoption and implementation feasibility, and digital monitoring contributed to maintenance and scalability. The evaluation structure consists of three primary domains, with specific instruments and outcomes summarized in Table 2 (Multidimensional Evaluation Tools for HOPE-FIT Program Based on the RE-AIM Framework).

Quantitative Measures

**Quality of Life**
  - EuroQol five-dimension, five-level scale (EQ-5D-5L).
  - World Health Organization Quality of Life–Brief (WHOQOL-BREF).
  - Selected for their validation in older adult populations and sensitivity to physical and psychosocial changes.

**Autonomy**
  - Basic Psychological Needs Satisfaction Scale, assessing autonomy, competence, and relatedness, which are essential for sustainable engagement.

**Social Support**
  - Multidimensional Scale of Perceived Social Support (MSPSS), evaluating perceived support from family, friends, and significant others.

**Exercise Adherence**
  - Exercise Adherence Rating Scale (EARS), validated for monitoring sustained participation in physical activity intervention

**Motivation and Participation**
  - Exercise Self-Regulation Questionnaire, capturing both intrinsic and extrinsic motivational factors that shape long-term adherence.

**Physical Function Tests** (administered with rest breaks and safety monitoring to ensure feasibility for older adults)
  - Body composition: Bioelectrical impedance device (InBody).
  - Lower body strength: 30-s sit-to-stand test with arms crossed.
  - Upper body strength: 30-s dumbbell curl (dominant hand, seated).
  - Lower body flexibility: Sit-and-reach test.
  - Upper body flexibility: Back scratch test.
  - Cardiovascular endurance: 2-min step-in-place test.
  - Dynamic balance: 2.44-m timed up-and-go test.

**Muscle Strength**
  - Isokinetic quadriceps and hamstring strength using a Cybex dynamometer for objective muscular assessment.

**Cognitive Function**
  - Mini-Mental State Examination (MMSE): general screening.
  - Korean version of the Montreal Cognitive Assessment (MoCA-K): detection of mild cognitive impairment.
  - Trail Making Test (TMT) A and B: attention, processing speed, and executive function.
  - Clock Drawing Test (CDT): visuospatial construction and planning ability.
  - Geriatric Depression Screening Questionnaire (GDSQ): depressive symptoms and emotional status.

**Digital literacy and Behavioral Economics Measures**
  - Digital literacy assessment.
  - Technology acceptance and perceived ease of use.
  - Survey of responsiveness to behavioral economic incentives (e.g., branded T-shirts, eco-bags, honoraria).

These measures were included to evaluate digital adaptation and incentive responsiveness, both essential for long-term maintenance and scalability of hybrid interventions.

**Smart Device-Based Biometric Indicators**
  - Sleep patterns, heart rate, gait speed, and EEG-based neurofeedback data collected via smart rings, radar sensors, and environmental detectors.

**Figure 7:**
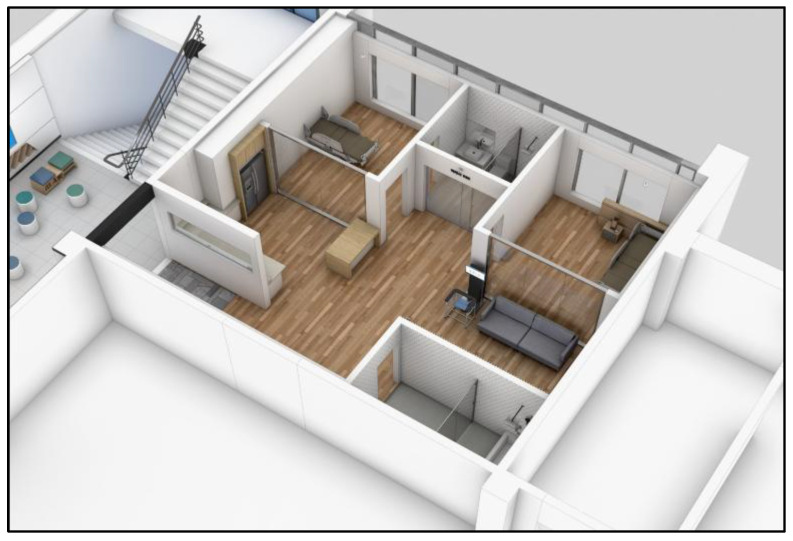
Photograph of smart-home health system installation. Photograph of the installation process for the smart-home health monitoring system. The system integrated wearable sensors, radar-based gait detectors, and sleep/heart-rate monitors to provide continuous data streams for ecological validity and safety oversight.

**Figure 8:**
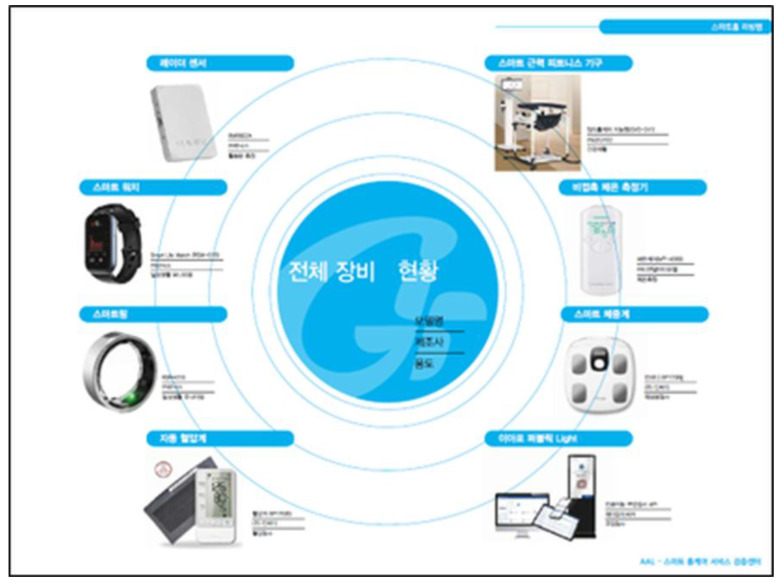
Inventory and setup of smart-home monitoring equipment. Inventory and setup of smart-home monitoring equipment used in the HOPE-FIT program. The figure displays the variety of research-grade devices, including smart rings, radar sensors, and EEG-based tools, prepared for deployment across participant households.

These systems enabled real-time monitoring for ecological validity and safety (see Figure 7 and Figure 8).

In addition to structured measurement points (baseline, post-intervention, and six-month follow-up), formative evaluation was embedded. For example, if ≥20% of participants reported new knee pain flags within one week, the HUB automatically triggered SHI triage calls, the substitution of a low-impact module, and healthcare provider audits. This real-time feedback loop was repeatedly used across project years (2021–2024), linking biometric and survey data to immediate protocol adjustments. Such iterative refinement reinforced the Hybrid Type II design and enhanced ecological validity.

### 3.5. Implementation Facilitators and Barriers

During the implementation of the HOPE-FIT model, several practical challenges emerged that require careful consideration for future scaling. One key difficulty lay in building trust with older adults when introducing smart-home installations. While the model included free provision and setup of devices such as sensors and monitoring systems, many participants expressed hesitation and discomfort with allowing outsiders into their homes, raising concerns about potential fraud or misuse. In practice, this required smart-home healthcare instructors (SHIs) and healthcare providers (HCPs) to dedicate additional time to explanation, reassurance, and step-by-step familiarization to ensure that participants fully understood the purpose and safety of the technology.

Another barrier was related to the installation of radar sensors for gait monitoring. Some older adults perceived the sensor as a form of unwanted exposure or intrusion, leading to resistance or outright refusal to have such devices installed in their living spaces. This highlighted that, in addition to technical feasibility, the perceived invasiveness of health technologies and privacy sensitivities must be addressed through culturally sensitive communication, transparent explanations of data use, and the provision of opt-out options.

At the same time, several facilitators supported implementation. The free installation and maintenance of devices, the presence of trusted SHIs who provided personalized guidance, and the use of gradual orientation sessions helped reduce anxiety and increase acceptance. Peer recommendations within community centers further enhanced credibility and engagement. Ethical safeguards including informed consent, institutional review board (IRB) approval, and secure data storage protocols reinforced participant trust.

To minimize potential bias arising from the same team being responsible for design, implementation, and evaluation, we established an independent monitoring system. This system includes external audits of implementation fidelity by experts unaffiliated with the intervention team, as well as oversight by an advisory board comprising academic, clinical, and community representatives. Importantly, more than 15 independent institutions—including physicians from partner hospitals, affiliated university medical centers, and external monitoring agencies—are engaged in auditing, data validation, and ethical review. These mechanisms ensure objectivity, enhance transparency, and strengthen trust in the replicability of the HOPE-FIT model.

Taken together, these experiences highlight that the successful implementation of hybrid health models such as HOPE-FIT depends not only on technical readiness but also on building relational trust, ensuring ethical safeguards, and tailoring communication strategies that honor autonomy and comfort. By anticipating barriers and actively leveraging facilitators, the model is more likely to achieve acceptance, adherence, and long-term sustainability in diverse community settings.

## 4. Data Collection Procedures

This study employed an integrated data collection framework to evaluate the effectiveness and feasibility of the HOPE-FIT-based SAGE program. Data were collected at three time points (pre-, mid-, and post-intervention) over a 12-week intervention period, incorporating quantitative, qualitative, and sensor-based measures. The procedures are detailed below.

### 4.1. Pre-Intervention Assessment

At the point of enrollment, all participants underwent a baseline assessment conducted either in person or remotely, depending on digital literacy and accessibility.

- Quantitative measures—such as quality of life, autonomy, and exercise adherence—were collected via structured questionnaires or app-based self-reports.
- Physical function was directly assessed by certified exercise specialists (ES) or physical therapists (PT) at the site.
- Cognitive function was evaluated in collaboration with neuropsychological experts using MMSE-K, MoCA-K, TMT (A&B), CDT, and GDSQ.
- For participants in the smart-home group, physiological data including heart rate, sleep patterns, stress levels, and gait characteristics were collected in advance using wearable smart rings, radar sensors, and EEG devices.

To minimize baseline bias, participants’ pre-existing knowledge and attitudes toward digital technologies were explicitly measured using a Digital Literacy Scale and Technology Acceptance Questionnaire. These variables are included as covariates in the analysis to adjust for their potential influence on self-reported measures of digital literacy and exercise adherence.

### 4.2. Mid-Intervention Monitoring

At Week 3, a mid-intervention assessment was conducted to monitor program retention, self-directed adherence, and engagement with digital tools.

- Evaluation tools included the Exercise Adherence Rating Scale (EARS), open-ended questionnaires on hybrid exercise experiences, self-directed activity logs, and app usage analytics.
- Participant engagement was indirectly monitored through digital communication platforms (e.g., app messaging) or follow-up inquiries.
- Selected key performance indicators (KPIs)—such as functional improvement and the digital compliance rate—were regularly tracked.
- Participant responses to each ACT session were gathered via post-session feedback forms and researcher observation notes, and behavioral-economics-based incentive reactions were concurrently assessed.

Self-reported data were triangulated with objective measures: for instance, app-based exercise logs were cross-validated with wearable-derived metrics (e.g., step counts, gait data, heart rate variability). Discrepancies between self-report and sensor-based data were flagged and reviewed during the quality improvement (QI) cycle, ensuring the reliability of the adherence measurements.

### 4.3. Post-Intervention Assessment

Upon completion of the 12-week intervention, a post-assessment identical to the baseline was conducted to evaluate program outcomes.

- All quantitative instruments were re-administered using the same modalities.
- For the qualitative evaluation, semi-structured interviews were conducted with 30 participants, covering topics such as exercise experiences, acceptance of smart-home technology, psychological changes based on ACT principles, and perceived social connectivity.
- Sensor-based data (EEG, smart rings, radar sensors) were collected within three days post-intervention and uploaded to the central HUB system. The system was configured for real-time alerts in cases of abnormal findings.
- These datasets were integrated into a personalized intervention algorithm and contributed to the quality improvement (QI) cycle for future program refinement.

### 4.4. Data Management and Ethical Considerations

- All collected data were securely stored in encrypted formats, with the anonymization of personally identifiable information.
- At each data collection point, participant consent was reaffirmed based on prior IRB approval.
- Qualitative data, including interview transcripts and open-ended responses, underwent double-coding and cross-validation to ensure analytic trustworthiness.
- After study completion, all data were centrally archived in the HUB server and incorporated into the next-stage intervention design through the QI cycle.
- To address privacy concerns related to commercial smart-home technologies, the program used research-grade devices operating under strict data-use agreements. No data were shared with manufacturers for advertising purposes. All sensitive health data were encrypted in transit and at rest, accessible only to the study team. Specific protocols for data security and participant confidentiality were established in compliance with national data protection regulations and IRB guidelines.
- In addition, independent data monitoring and external auditing processes are implemented. More than 15 collaborating institutions—including university hospitals, external research centers, and independent monitoring bodies—review data integrity, verify analytic procedures, and ensure fairness in evaluation. These external reviews are scheduled periodically and function as an added safeguard to reduce bias and uphold transparency throughout the study.

## 5. Dissemination Plan

The HOPE-FIT model and SAGE program were designed as a representative example of a hybrid approach aimed at the multidimensional enhancement of older adults’ well-being. By integrating medical, exercise, psychological, and community-based elements into a comprehensive health management system, the model seeks to establish a scalable, equity-driven framework. Rather than focusing on a single implementation site, the current study adopts a multilayered dissemination strategy to catalyze scholarly, clinical, and policy-level diffusion with real-world impact.

(1) Academic Dissemination Strategy

This study aims to foster transdisciplinary collaboration across the domains of aging, public health, digital healthcare, and implementation science. To this end, the research team plans to organize international academic symposia and actively engage in interdisciplinary research networks. Through invited sessions, joint workshops, and policy-relevant panel discussions—co-hosted in partnership with leading global institutions—the project will enhance scholarly influence and amplify its translational messaging. Such efforts will position the HOPE-FIT model not merely as an intervention tool, but as a meta-model that is applicable across diverse academic and institutional environments.

(2) Policy and Stakeholder Engagement Strategy

The core findings of this study will be repackaged into summary reports and policy briefs for dissemination to public health centers, local governments, national ministries, and relevant public agencies. Specifically, the study will offer practical proposals on how the HOPE-FIT model can be effectively integrated into national health promotion frameworks, such as community-integrated care, smart health pilot initiatives, and digital inclusion policies. These proposals will serve as foundational material for implementation guidelines, ultimately contributing to greater health equity and policy acceptance at the systemic level.

(3) Community and Clinical Dissemination Strategy

The HOPE-FIT and SAGE programs were originally conceived as community-based interventions. In collaboration with smart health enterprises, community centers, and healthcare institutions, the model will be adapted to suit varying local needs and conditions. Implementation manuals, training materials, and digital toolkits will be systematically developed and distributed to ensure fidelity, acceptability, and sustainability. Moreover, these educational resources will be linked to formal certification programs accredited by relevant professional associations, facilitating the development of implementation capacity.

(4) Digital and Open Science Dissemination Strategy

Core resources—such as infographics, intervention protocols, and instructional videos—will be gradually made available through the Wello! platform and official institutional repositories. However, given the complexity of the multi-year intervention structure and the sensitivity of physiological and psychological data from high-risk populations, the project will avoid fully open platforms such as Zenodo or OSF. Instead, it will establish a secure, credential-based sharing ecosystem. Anonymized datasets and codebooks will be selectively shared with certified researchers and partner institutions, under formal agreements, to enable follow-up analysis and replication. This approach balances data security with scientific transparency and may serve as a model for improving digital access among underserved populations.

(5) Dissemination Vision Grounded in Social Value

This study begins with a fundamental question: “Who wouldn’t want to age naturally?”. However, when anti-aging technologies and smart health solutions proliferate through high-cost markets, the right to healthy aging risks becoming a privilege for the few. The HOPE-FIT model offers a cost-effective and inclusive approach that promotes physical, emotional, and digital empowerment without the need for expensive equipment or high-intensity treatments. Rather than just delivering a program, this dissemination strategy serves as a practice-based solution to expand the possibility of healthy and dignified aging for all—including socially marginalized and digitally excluded populations.

## Figures and Tables

**Table 1:** Integrated evaluation matrix based on the RE-AIM framework and Hybrid Type II Design.

RE-AIM Element	Evaluation Domain	Hybrid Design Type	Evaluation Indicators and Application
Reach	To what extent the program reached a broad target population	Implementation	Number of participants and recruitment rate Diversity of participant characteristics (age, gender, socioeconomic background, etc.) Digital accessibility and presence of excluded individuals
Effectiveness	Whether the intervention led to clinically and behaviorally significant changes	Effectiveness	Changes in physical activity levels Improvements in functional fitness Quality of life (QoL) Self-reported health indicators Degree of social participation
Adoption	Whether organizations and practitioners adopted and delivered the program	Implementation	Number of participating institutions Types of institutions (e.g., welfare centers, public health centers) Program adoption rate Participation rate of professionals (e.g., exercise, medical experts)
Implementation	The extent to which the program was delivered as intended	Implementation	Fidelity of session delivery Timeliness of feedback Utilization rate of smart technologies (e.g., sensors, rings) Training completion rate and level of interdisciplinary collaboration
Maintenance	Sustainability of program outcomes and implementation over time	Effectiveness + Implementation	Maintenance of physical activity and health indicators at six-month follow-up Willingness of participants to re-engage Plans for continued implementation by institutions Foundation for long-term follow-up study design

**Table 2:** Multidimensional evaluation tools for the HOPE-FIT program based on the RE-AIM framework.

Evaluation Domain	Measurement Tools or Description
Quality of Life (QOL)	EQ-5D-5L, WHOQOL-BREF
Autonomy	Basic Psychological Needs Satisfaction Scale
Social Support	Multidimensional Scale of Perceived Social Support (MSPSS)
Exercise Adherence	Exercise Adherence Rating Scale (EARS)
Exercise Motivation/Participation	Exercise Self-Regulation Questionnaire
Physical Function	30-s Chair Stand, 2-Minute Step Test, Single Leg Stance, Sit and Reach, Back Scratch Test
Muscle Strength Assessment	Isokinetic strength test for quadriceps and hamstrings using a handheld dynamometer
Cognitive Function Assessment	MMSE, MoCA-K, Trail Making Test (TMT) A & B, Clock Drawing Test (CDT), Geriatric Depression Scale Questionnaire (GDSQ)
Digital Literacy and Behavioral Economics	Digital skills, technology acceptance, incentive-related perceptions survey
In-depth Interviews	Semi-structured interviews on program experience and perceptual changes
Open-ended Questionnaire	Qualitative responses on exercise perception, enjoyment, recovery, and community engagement
Session-based ACT Evaluation Sheet	Evaluation of acceptance, emotional awareness, value clarification, and committed action
Values-based Commitment Questionnaire	Changes in value-oriented life direction and behavioral commitment
Wearable Devices	Real-time monitoring of heart rate, sleep quality, and stress index using smart rings
Environmental Sensors and IoT Devices	Gait patterns, fall detection, and sleep behavior via radar and ambient sensors
EEG (Electroencephalography)	Quantitative analysis of mental immersion, stress response, and attention (e.g., α/β wave ratio)

## Data Availability

The data presented in this study are not publicly available due to the multi-year nature of the research, its integration with government-supported initiatives, and collaborative agreements with multiple partner institutions. The dataset includes sensitive operational records, digital biomarker logs, and implementation metrics tied to regional health infrastructure and smart home platforms. Therefore, it is not feasible to share the raw data. However, further information on the study’s infrastructure—including the Smart-Home Health Monitoring System, community-based exercise sites, and the affiliated Institute for Integrated Exercise Rehabilitation Research—is available via the following resources: Smart Home Platform: https://aal-hec.co.kr/ (accessed on 17 June 2025); Research Sites Overview: http://www.crflab.co.kr (accessed on 17 June 2025); While the raw data cannot be publicly disseminated, the authors warmly welcome academic inquiries and collaborative research opportunities. Interested researchers are encouraged to contact the corresponding author for further discussion. Suyoung Hwang, Email: harriett0059@gmail.com.

## Abbreviations

The following abbreviations are used in this manuscript:

HOPE-FIT	Hybrid Outreach Program for Exercise and Follow-up Integrated Training
SAGE	Senior Active Guided Exercise
ACT	Acceptance and Commitment Therapy
ACPT	Acceptance and Commitment Performance Training
RE-AIM	Linear dichroism
HCP	Healthcare provider
SHI	Smart home healthcare instructor
ES	Exercise specialist
OT	Occupational therapist
PT	Physical therapist
NT	Nutritionist
DH	Dental hygienist
HP	Health psychologist
UI/UX	User interface/user experience
EEG	Electroencephalography
MOCA-K	Montreal Cognitive Assessment—Korean version
MMSE-k	Mini-Mental State Examination—Korean version
TMT	Trail Making Test
CDT	Clock Drawing Test
tVNS	Transcutaneous vagus nerve stimulation
